# Supplementary material for: Impact of functional synapse clusters on neuronal response selectivity
Source: Nat Commun. 2020 Mar 16;11:1413. doi: 10.1038/s41467-020-15147-6 (PMC7075899; doi:10.1038/s41467-020-15147-6)
Supplement: Supplementary file 1 — Supplementary Information [file 41467_2020_15147_MOESM1_ESM.pdf]

Impact of functional synapse clusters on neuronal response selectivity

Balázs B Ujfalussy[1,*], Judit K Makara[1]

[1] Laboratory of Neuronal Signaling, Institute of Experimental Medicine, 1083 Budapest, Hungary
[*] email: balazs.ujfalussy@gmail.com

Supplementary Figures

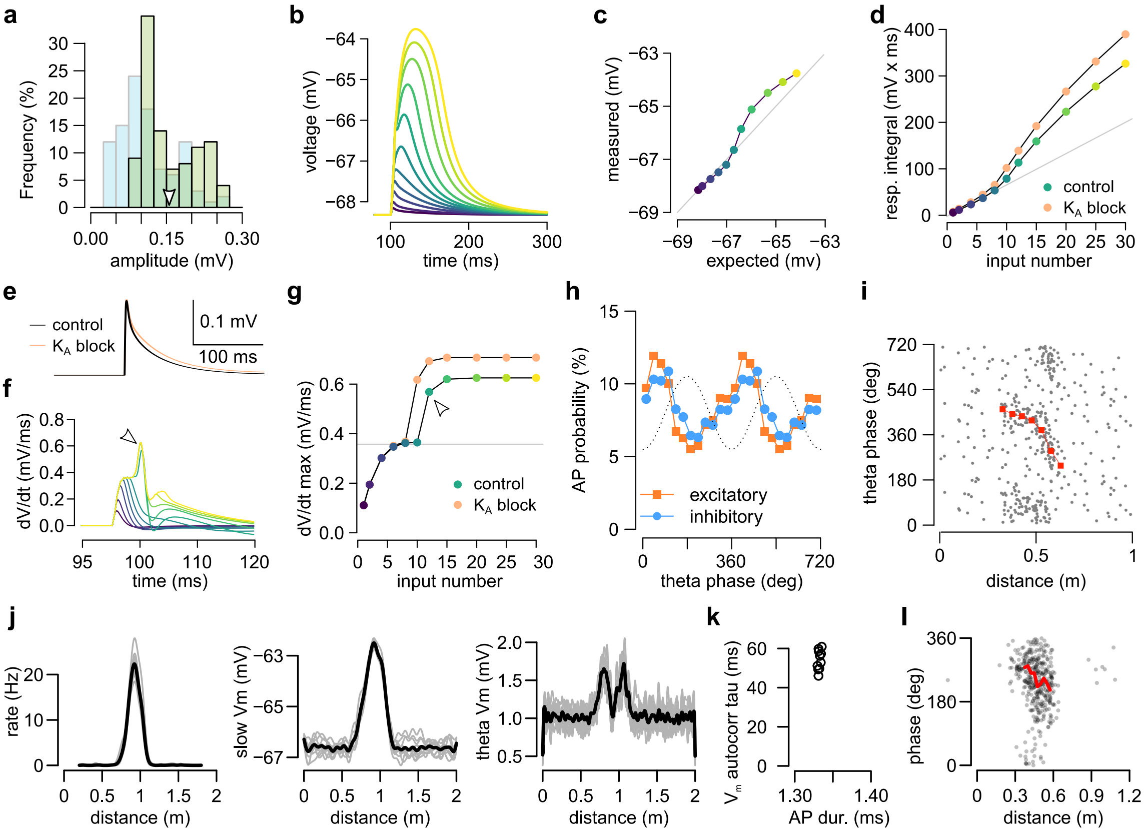

Supplementary Figure 1. Dendritic integration, input statistics and somatic membrane potential of the model CA1 neuron. a-g Dendritic integration. **a** Histogram of the somatic EPSP amplitudes. Total 100 synapses were distributed randomly along the apical trunk at 50-300 μm distance from the soma (green, directly comparable to experimental data in ref. [1]) or along the entire dendritic tree (light blue). Synapses were activated individually. Arrowhead indicates the mean of the trunk synapse amplitudes. **b** Somatic membrane potential in response to the activation 1-30 synapses on an oblique branch with δt=0.3 ms. Color code is the same as in D. **c** Measured response amplitude as a function of linear expectations. Color code is the same as in D. **d** The integral of the somatic response as a function of the number of stimuli. Grey line shows linear expectation fitted to the first 5 datapoints. Orange dots show that the nonlinearity was larger when A-type K^+ channels were blocked. The threshold for NMDAR nonlinearity was around 10 stimuli. **e** Example of the somatic response to the activation of a single synapse in control conditions (black) and under partial blockade of dendritic A-type K^+ channels. A-type potassium channels regulate the duration of EPSPs but not their amplitude. **f** Derivative of the somatic responses shown in panel b indicating the presence of local Na^+ spikes for sufficiently strong stimuli (arrowhead pointing to the spikelets). Color code is the same as in D. **g** The peak amplitude of the derivative of the somatic response showed a step-like increase when local Na^+ spikes were generated (arrowhead). The threshold for dendritic Na^+ spikes was similar to the threshold for NMDAR nonlinearity (D). The amplitude of the somatic dV/dt was increased when A-type K^+ channels were blocked (orange). **h-i** Input statistics. **i** Theta modulation of inhibitory (blue) and excitatory inputs (orange) to the CA1 model neurons during the theta state (cf., Fig 2J of ref. [2]). **i** Phase precession of an example excitatory input in 16 trials. Grey dots indicate single spikes and red symbols show circular mean phase. Note that each spike is shown twice at ϕ and $\phi + 360°$. Also note that the high background firing outside the place field accounts for the activity of ~10 cells not having place field on this track (cf. Fig. 7 of ref. [3]). **j-l** Somatic membrane potential statistics in the LTP case without clustering (c.f., Fig. 3). **j** Firing rate, slow V_m and theta V_m as the function of position for 10 input arrangements (grey) and the average (black) (cf. Fig. 2E of ref. [2]). Note that experimental data are typically reported without correction for the liquid junction potential (~ 8 mV). After correction, the V_m values in the model correspond well to experimental results. **k** Membrane potential autocorrelation time constant and action potential half width at half maximum for 10 arrangements (cf. Fig. 2D of ref. [2]). **l** Output spike theta phase as the function of the position for an example input arrangement. Red line indicates circular mean (cf. Fig. 7D of ref. [2]).

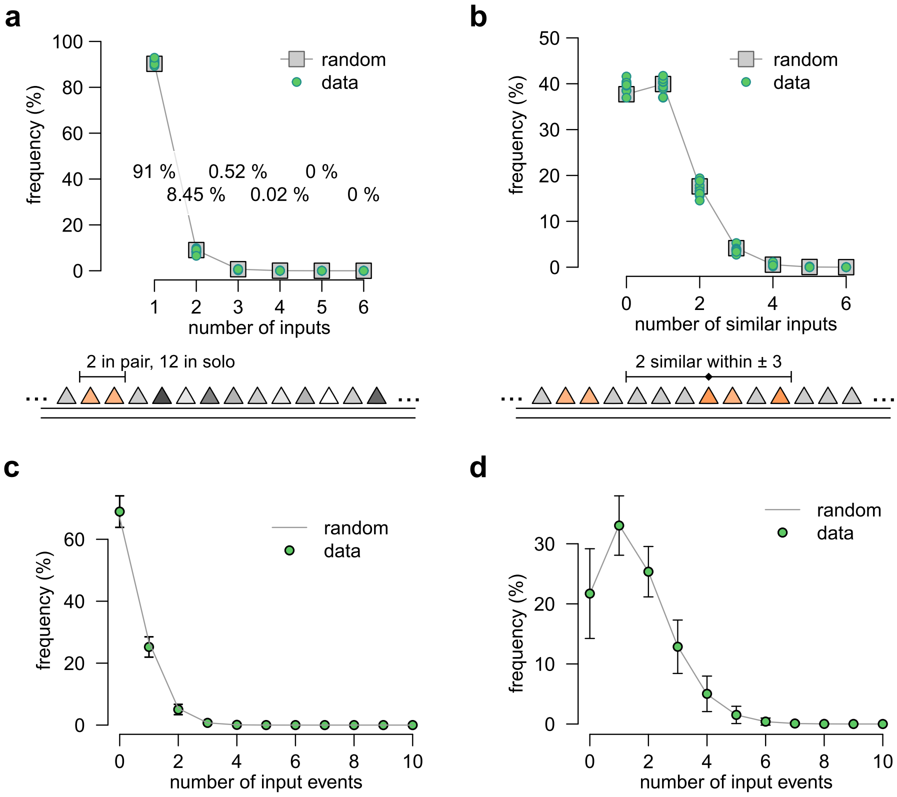

Supplementary Figure 2. Synaptic clusters with random connectivity. a Distribution of the number of neighbouring inputs along the postsynaptic dendritic branch of the biophysical model (inset at the bottom) with identical place field location (green, n=10 different configuration patterns). The data is consistent with a pattern expected from random innervation (grey) and indicates that $\sim 8\%$ of the inputs occurred in pairs but larger clusters contained less than 1% of the inputs. **b** Distribution of the number of inputs with similar tuning within the 6 neighbouring inputs (inset at the bottom). Here we used a looser measure of functional clustering than in panel a: First, similarly tuned inputs could be separated with unrelated inputs. Second, we allowed a 15 cm distance between the peaks of the presynaptic place fields. Even with this less conservative definition of functional synaptic clustering, clusters of 4 synapses were rare. **c** Distribution of the number of synaptic events arriving at 100 μm long segment of a single dendritic branch within 10 ms during theta state. With random connectivity the typical number of input rarely exceeded 2 spikes/10 ms. **d** Same as panel c during SPWs, when the typical input could be as large as 4-5 spikes per 100 μm dendrite within 10 ms. Symbols in panels c-d show mean and SD across $n = 1920$ repetitions (12 dendritic branches, 16 synaptic configurations and 10 independent simulations each).

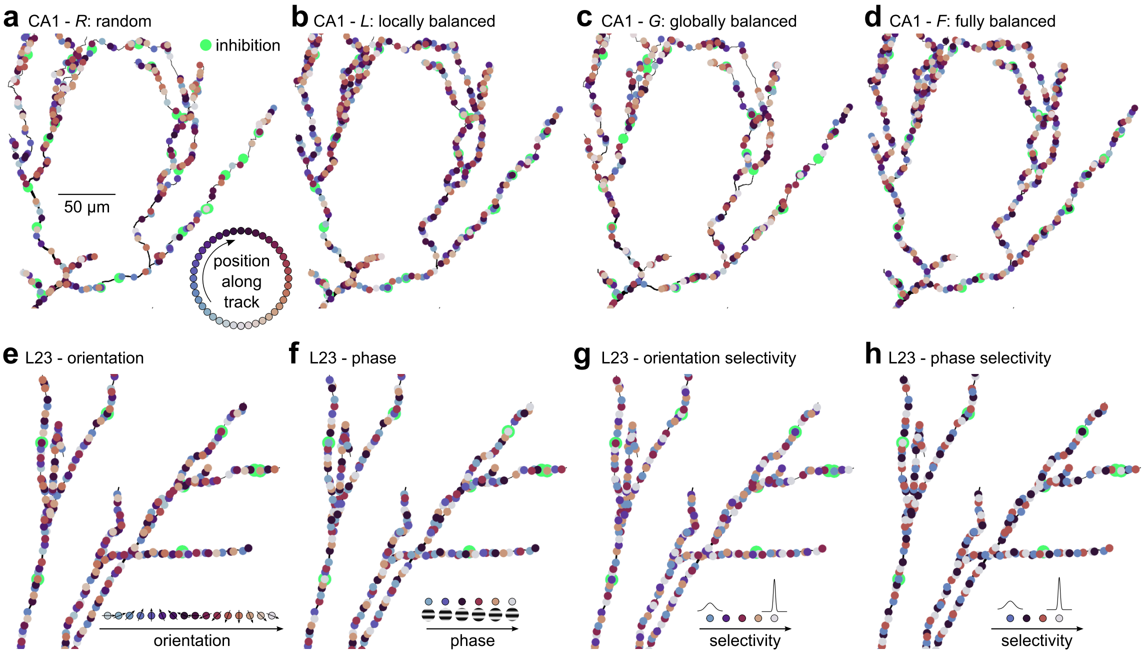

Supplementary Figure 3. Connectivity patterns in hippocampal CA1 and visual cortical L2/3 cells. a-d Connectivity patterns in hippocampal CA1 neuron. **a** Example for random connectivity in a CA1 subtree. Colors indicate place field position, with distance in the color space matching distance between place field locations in a circular track (inset). Large green circles indicate inhibitory inputs. Synapse locations are approximate. **b** Example for locally random, globally balanced connectivity. Synapses were first ordered globally into a single sequence mapped on the dendritic tree minimizing both small-scale functional clustering and large scale inhomogeneities, as in panel d. Next, synapses were permuted and their location were randomized within each branch, potentially generating small scale functional clustering but not large scale biases. **c** Example for globally random, locally balanced connectivity. Synapses were first arranged randomly throughout the entire dendritic tree. Next, synapses within each branch are organised into a single sequence to remove small-scale functional clustering while retaining large scale biases. **d** Example for fully balanced connectivity. Synapses were ordered globally into a single sequence mapped on the dendritic tree minimizing both small-scale functional clustering and large scale biases. **e-h** The fully balanced connectivity in visual cortical L2/3 cell. Inputs in the visual cortex were higher dimensional as they were tuned to both orientation and phase and had different selectivity (i.e., simple cells had a narrow phase tuning while complex cells were widely tuned). Inputs were organized in an approximately balanced way with respect to all four variables. Color code indicates the tuning of the cells with respect to the $N_\theta = 16$ different orientation preference (E), $N_\varphi = 6$ different phase preference (F), $N_\Theta = 5$ different orientation selectivity (G) and $N_\Phi = 4$ different phase selectivity (H). As $\{N_\theta, N_\varphi, N_\Theta, N_\Phi\}$ were not co-prime numbers, we could not use independent co-prime reordering to achieve a coherent rearrangement of $N_\theta \times N_\varphi \times N_\Theta \times N_\Phi = 1920$ inputs. **e** We used co-prime reordering of the 16 orientation preference classes using $\alpha = 5$. The same orientation-preference sequence was repeated 15 times (240 inputs) after which the base sequence was shifted by 3 for the next 15 repeats followed by further 15 repeats with shifts $\{6, 1, 4, 7, 2, 5\}$. **f** The sequence $\{1, 3, 5, 2, 4, 6\}$ was repeated 320 times. **g** The sequence $\{1, 4, 2, 5, 3\}$ was repeated 384 times. **i** The sequence $\{1, 3, 2, 4\}$ was repeated 15 times and $\{3, 2, 4, 1\}$ was repeated 15 times and then the whole sequence was repeated 16 times.

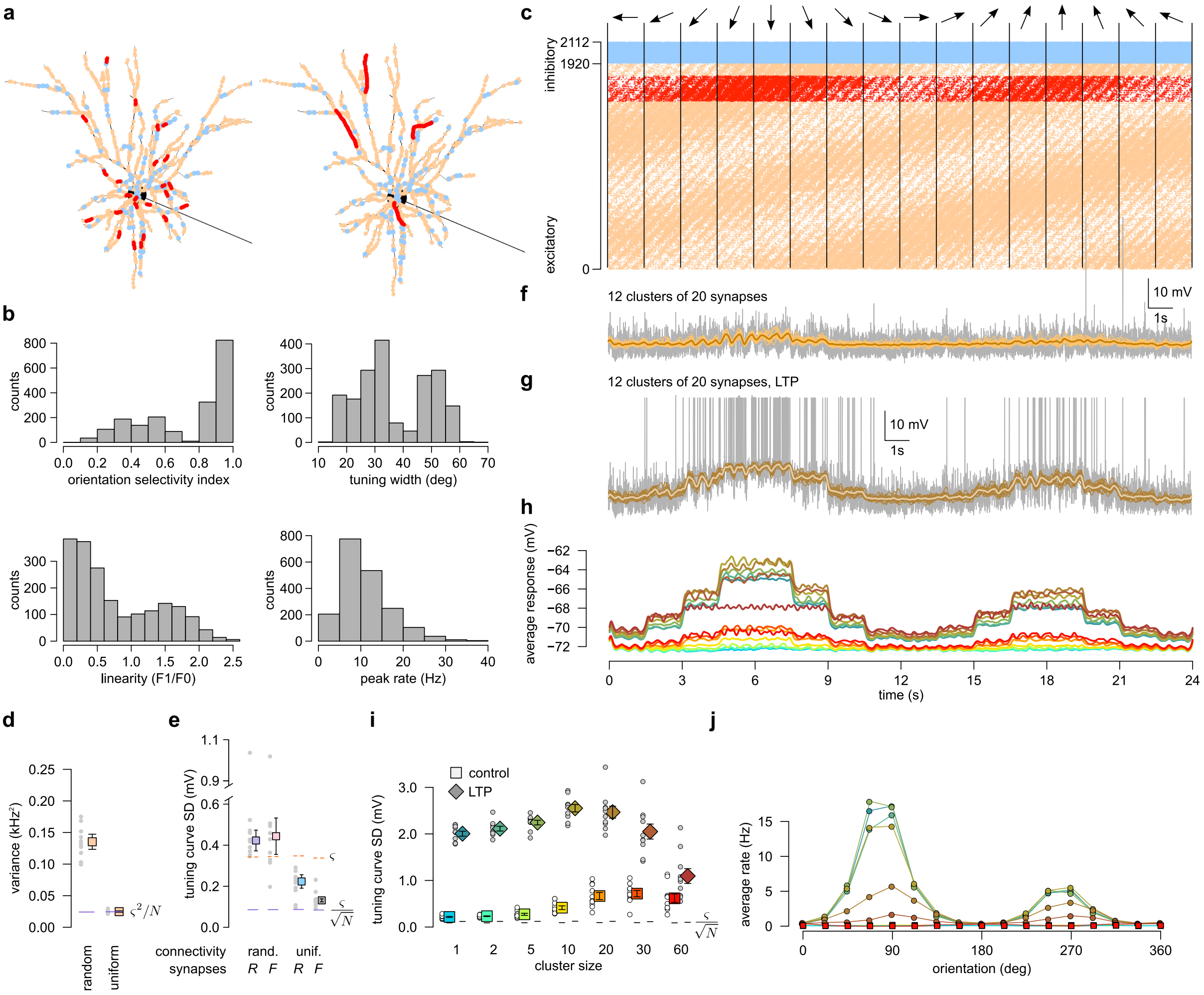

Supplementary Figure 4. Effect of functional synaptic clusters in L2/3 neurons. **a** Arrangement of the 240 synapses (red) into 24 (left) or 4 (right) functional synaptic clusters together with the 1680 background excitatory (orange) and 192 inhibitory (blue) synapses on the model L2/3 neuron. **b** Distribution of orientation selectivity (top left), tuning curve width (top right), receptive field linearity (bottom left) and the peak firing rate (bottom right) of the inputs. Receptive field linearity was defined as the ratio between the amplitude of the oscillatory component of the response (F1) and the mean response (F0) to the preferred stimulus[4]. For comparable experimental data, see Fig. 4A, 4C 7A, and 8E of ref. [4], respectively. **c** Activity of excitatory (orange and red) and inhibitory (blue) inputs in response to simulated drifting gratings in 16 different orientations (arrows on the top), each shown for 1.5 s. Excitatory inputs were ordered according to their direction preference. A subset of the inputs responded to movements in the opposite directions (orientation selectivity) leading to the second, weaker band of spikes. Excitatory inputs preferring the down direction were chosen for clustering (red). **d** Variability of the inputs. The variance of the mean filtered spike count was higher than (same as) the variance expected from trial-to-trial variability (ς^2/N, solid line) when inputs were random (uniform), respectively. Symbols indicate mean±SEM. **e** Tuning curve standard deviation (SD) with random (R) and fully balanced (F) connectivity and with random or uniform inputs in the absence of systematic functional clustering. Purple line segments show the SD expected based on trial-to-trial variability ($\sqrt{\varsigma^2/N}$) and orange dashed lines show trial-to-trial SD (ς). Since visual cortical inputs are tuned to multiple features, we could not entirely eliminate the effect of dendritic processing on the tuning curve even in the fully balanced connectivity (tuning curve SD is larger than $\sqrt{\varsigma^2/N}$ even in the uniform, fully balanced case). Symbols in this panel show median and quartiles as data is highly non-Gaussian (note the outliers). **f** Example sVm response of the L2/3 neuron (grey), slow V_m for 16 trials (orange) and the tuning curve (brown) with 12 clusters of 20 synapses. **g** Same as d with potentiated clustered synapses. **h** Average subthreshold response of the L2/3 postsynaptic cell showed an increased depolarisation amplitude with clustering. Potentiation of the clustered synapses further increased the response amplitude. Color code is the same as in panel i. **i** Tuning curve SD as a function of synaptic clustering for control (bright colors) and LTP (dark colors) in 10 independent simulations (circles) and their means (color boxes) and SEM (error bars). Note that in e and i we show tuning curve SD instead of the variance (as in Fig. 3.), since the variance would further exaggerate the already large differences between control and LTP. **j** Firing rate of the postsynaptic L2/3 cell as a function of orientation with different levels of clustering. Color code is the same as in panel i. The firing rate was near zero in all cases without LTP.

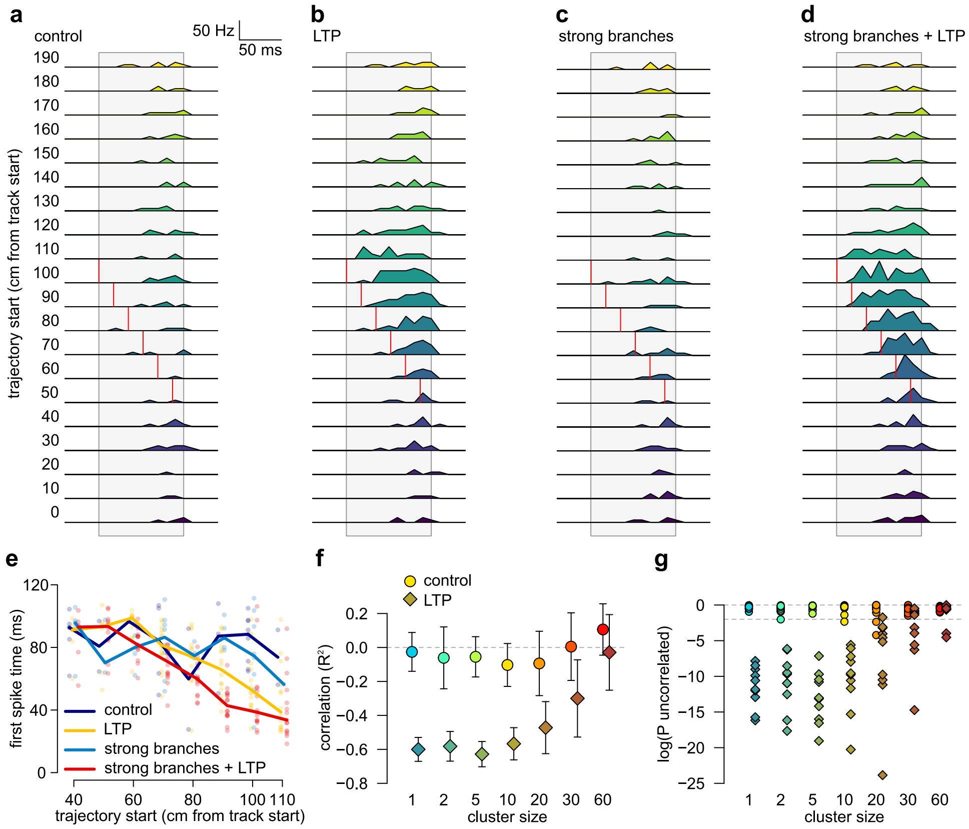

Supplementary Figure 5. Spike timing during sharp waves. a-b Average firing rate during SPWs in control (a) and with LTP (b) with 10 synapses per cluster. Different lines show different replayed input trajectories with the start location of the trajectory indicated on the left. The duration of the SPW is highlighted by the grey box. Red line indicates the approximate activation of the clustered input synapses within the input trajectory (see also Figure 4a). **c-d** Same as a-b with strongly excitable dendritic branches without (c) and with (d) LTP. **e** First spike timing as function of trajectory start for the data shown in a-d. Colored dots: data from 16 individual trials (not all of them contained action potential), lines: median. The correlation between spike timing and trajectory start was R^2=-0.11, p=0.57 (control), R^2=-0.61, p=$2.6{\cdot}10^{-11}$ (LTP), R^2=-0.24, p=0.14 (strong branches), R^2=-0.75, p=$1.8{\cdot}10^{-21}$ (strong branches + LTP; Pearson's R^2 and p-value from Pearson's product-moment correlation test). **f** Correlation (Pearson's R^2) between trajectory start and first spike timing as a function of cluster size under control conditions and in the presence of LTP. Symbols show mean and SD across 10 different cluster arrangements. (Data with strongly excitable branches is not shown since we could not generate 10 different cluster arrangements involving independent strong branches - see Supplementary Figure 6a.) **g** Significance of the correlation (p-value from Pearson's product-moment correlation test) as a function of cluster size for control and LTP. Symbols indicate individual datapoints.

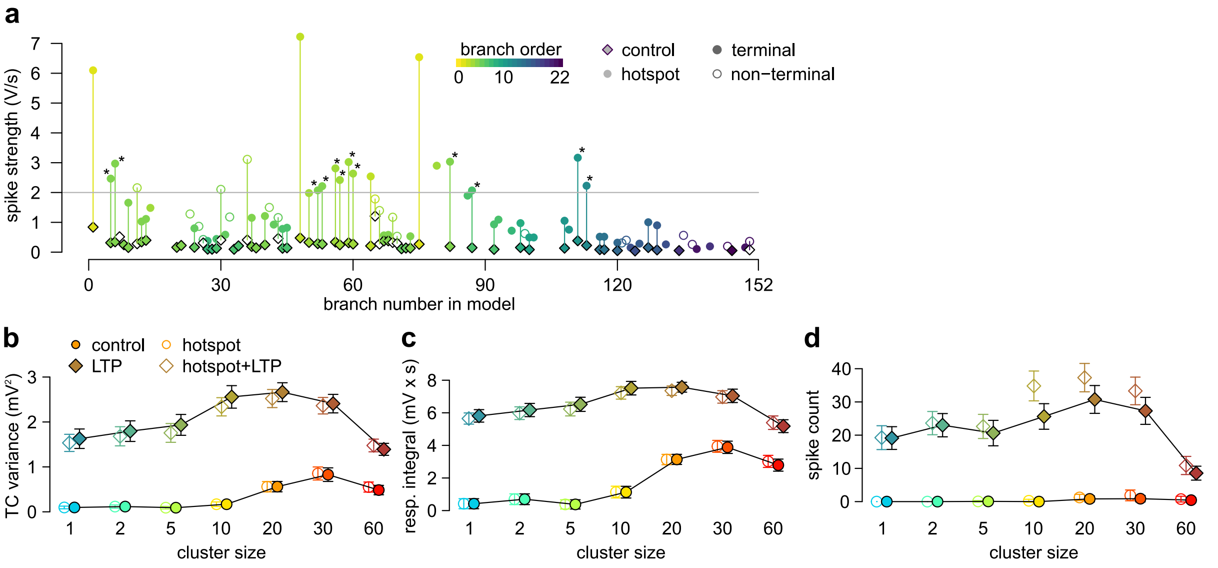

Supplementary Figure 6. Strong dendritic branches and their effect on clustering based tuning during theta activity. **a** Strength of the Na^+-spike in all dendritic branches with and without the hotspot. Color indicates the number of branch points between the stimulation site and the soma. Open circles indicate non-terminal branches. In some branches (e.g., 2-4) no local dendritic spike could be evoked. Twelve terminal branches longer than 60 μm were selected for adding hotspots for the simulations in panels b-d, in Figure 5 and in Supplementary Figure 5c-e, resulting in strong ($> 2\,\mathrm{V/s}$) dendritic Na^+ spikes. **b-d** The presence of hotspots (open symbols) only slightly influenced the tuning curve variance (b) and the response integral (c), while it increased the firing rate in the place field when LTP and large synaptic clusters were both present (d). Symbols show mean and variance of 16 trials with identical input configuration.

Supplementary References

[1] Magee, J.C. & Cook, E.P. Somatic EPSP amplitude is independent of synapse location in hippocampal pyramidal neurons. *Nat. Neurosci.* **3**, 895–903 (2000).

[2] Grienberger, C., Milstein, A.D., Bittner, K.C., Romani, S. & Magee, J.C. Inhibitory suppression of heterogeneously tuned excitation enhances spatial coding in ca1 place cells. *Nat. Neurosci.* **20**, 417–426 (2017).

[3] Skaggs, W.E., McNaughton, B.L., Wilson, M.A. & Barnes, C.A. Theta phase precession in hippocampal neuronal populations and the compression of temporal sequences. *Hippocampus* **6**, 149–72 (1996).

[4] Niell, C.M. & Stryker, M.P. Highly selective receptive fields in mouse visual cortex. *J. Neurosci.* **28**, 7520–36 (2008).
